# Age-related Smell and Taste Impairments and Vitamin D Associations in the U.S. Adults National Health and Nutrition Examination Survey

**DOI:** 10.3390/nu12040984

**Published:** 2020-04-02

**Authors:** Galya Bigman

**Affiliations:** Division of Epidemiology and Prevention, Institute of Human Virology, University of Maryland School of Medicine, Baltimore City, MD 21201, USA; gbigman@ihv.umaryland.edu or bigman.galya@gmail.com; Tel.: +1-512-576-3823

**Keywords:** smell impairment, taste impairment, serum 25-hydroxyvitamin D, Vitamin D, adults, NHANES

## Abstract

Smell and taste decline with aging, and markedly deteriorate when nutritional deficiencies occur. This study aims to examine the associations between Vitamin D (VD) deficiency and smell and taste impairments among adults. This paper details a cross-sectional study utilizing data from the US National Health and Nutrition Examination Survey (NHANES, 2013–2014.). Smell impairment was assessed by the Pocket Smell Test and defined as failing to correctly identify six or more of the eight odors. Taste impairment was defined as failing to correctly identify quinine or sodium chloride. VD was measured as serum 25-hydroxyvitamin. Multivariable weighted logistic regressions were utilized. Adjusted odds ratio (OR) and 95% confidence interval (CI) were presented. Overall, 2216 (smell sample) and 2636 (taste sample) participants were included, aged between 40 and 80 years old. Of those, 18.3% had taste impairment, 12.2% had smell impairment, and 20% had VD deficiency (<20 ng/mL). Compared to participants with sufficient VD (>30 ng/mL), those with VD deficiency were more likely by 39% to report a higher prevalence of smell impairment (OR = 1.39, 95%CI: 1.02–1.89); and only participants aged 70–80 years with VD inadequacy (20–30 ng/mL) were more likely by 96% to report a higher prevalence of taste impairment (OR = 1.96, 95%CI: 1.35–1.85). VD may have a significant role in age-related smell impairment in adults aged 40 years or older, and in age-related taste impairment in the elderly aged 70–80 years.

## 1. Introduction

Age-related smell loss alone or in combination with loss of taste can have negative influences on an individual’s food preferences, diet, and nutritional status and may impact the overall health condition [1]. Losses of smell and taste become a more common problem as people progress into adulthood and live longer and so pose a major risk to public health [2]. In the US, people over the age of 40 years have a 13.5% probability of smell dysfunction and a 17.3% probability of taste dysfunction. Taste and smell decline markedly with age, can affect up to 60% of individuals aged over 65 years and 80% of individuals aged over 80 years and over, and is more common in men than women [2,3,4].

Aging is one of the leading causes of smell loss due to cumulative damage to the olfactory receptor cells, ossification of the foramina of the cribriform plate, and changes in neural responsiveness [2]. Other causes include head injuries, illnesses such as cancer and infectious disease, environmental hazards such as insecticides, and medications (i.e., some antibiotics and antihistamines) [5]. Nutritional deficiencies also contribute to these deteriorations including trace metal deficiencies such as copper [6], zinc [7], and magnesium [8] and some vitamin deficiencies including Vitamin A [9], E [10], and B12 [11].

Vitamin D receptors are widely spread throughout the human brain and are involved in various brain neurotransmitters [12,13]. Vitamin D can cross the blood–brain barrier and bind to those receptors [14]. Mounting evidence has shown a relationship between Vitamin D deficiency and the nervous system, including impaired brain development and cognitive performance, as well as stroke, dementia, multiple sclerosis, Parkinson’s disease and epilepsy [15,16].

Furthermore, the ubiquitous presence of Vitamin D receptors was detected in the olfactory system of the rat [17], which has some striking similarities to the human olfactory system [18]. Other animal studies showed that Vitamin D influences neural stem cells and progenitor cell proliferation [19], creating various neuron types in the brain [20] and olfactory system [21]. Evidence on Vitamin D associated with the human olfactory system was recorded in one case report [22].

However, the role of Vitamin D on age-related smell and taste impairments among adults has never been examined in a large population-based study. Given this background, and to fill the void in knowledge, this study aims to examine the association between Vitamin D deficiency and smell and taste impairments and effect modification of age in the population of the US. This study hypothesizes that there are significant associations between Vitamin D deficiency and smell and taste impairments, being higher among older people.

## 2. Materials and Methods

### 2.1. Study Population

Data were utilized from the US National Health and Nutrition Examination Survey (NHANES) 2013–2014 conducted by the National Center of Health Statistics (NCHS) at the Centers for Disease Control and Prevention (CDC) [23]. The NHANES is a national population-based cross-sectional survey employing a stratified multistage probability design. Data on the health and nutritional status of non-institutionalized civilians of the US population are collected through a series of interviews, examinations and laboratory measurements. The NCHS Research Ethics Review Board (ERB) provided the following protocol approval number for the presented survey: Continuation of Protocol #2011–2017 (NHANES 2013–2014). Further information on the NHANES database is available at http://www.cdc.gov/nchs/nhanes.htm [23].

#### Study Sample

From the NHANES 2013–2014, participants who had available data on serum 25-hydroxyvitamin D, smell and taste assessments, and demographics were selected. In the smell sample, participants who reported problems with their nose such as sneezing frequently, discolored nasal mucus, nasal blockage, and sinus pain and those were not able to cooperate and understand the test were excluded from this examination and this sample. In the taste sample, participants with a quinine allergy or those who were not able to cooperate and understand the test were excluded from this examination and this sample. Finally, participants with missing data in one of the covariate variables were excluded. Therefore, the final smell sample included 2216 eligible participants from 2632 potential participants and the final taste sample included 2636 eligible participants from 3113 potential participants.

### 2.2. Study Variables

#### 2.2.1. Main Exposure

The major circulating form of Vitamin D in the serum is 25-hydroxyvitamin D (25(OH)D), which is considered the best indicator of Vitamin D from cutaneous synthesis and nutritional intake [24]. The NHANES used the liquid chromatography–tandem mass spectrometry (LC–MS/MS) method to measure serum 25(OH)D concentrations in the participant’s blood samples. The method has better analytical specificity and sensitivity compared to immunoassay methods, and fixed analytical goals for imprecision (≤10%) and bias (≤5%) [25]. To convert from SI units (nmol/l) to conventional units, nanograms per milliliter (ng/mL), serum 25(OH)D concentrations were divided by 2.4959 [25].

Serum 25(OH)D concentrations were classified according to Institute of Medicine criteria [26] as follows: < 20 ng/mL, at risk of Vitamin D deficiency; 20–30 ng/mL, at risk of Vitamin D inadequacy; >30 ng/mL, Vitamin D sufficiency [27,28]. Blood samples were collected during the period of November–April in southern US and during the period of May–October in northern US.

#### 2.2.2. Main Outcomes

##### Smell and Taste Assessments

The smell was assessed by the NHANES Pocket Smell Test (also known as the 8-item “scratch and sniff” test) based on the 40-item University of Pennsylvania Smell Identification Test (UPSIT) [29]. The test contained eight specific odorants that were released by scratching the test strips and presented to the individuals in a specified order: chocolate, strawberry, smoke, leather, soap, grape, onion, and natural gas. For each odorant, the participant smelled the odor and tried to identify it by choosing one answer from the four listed alternative options. Individuals who were incapable of correctly identifying six or more odorants out of the eight were categorized as having smell impairment.

The taste examinations included tongue tip taste and whole mouth taste testing [30]. The tongue tip taste testing included two tastings (1 mM quinine as a bitter taste and then 1 M NaCl as a salt taste) that were presented in a fixed presentation order at the tip of the tongue with a cotton swab applicator. Each participant was asked to identify it as salty, bitter, sour, some other taste, or no taste. The mouth was rinsed with water before proceeding to the subsequent tasting.

In the whole mouth taste testing, each participant was presented with three tastings (0.32 M NaCl, 1 mM quinine, and 1 M NaCl) in one of two randomized presentation orders. The participant was instructed to swish the 10 mL tasting solution in the mouth for three seconds, spit it out, and then provide a taste intensity and identify it as salty, bitter, sour, some other taste, or no taste. The mouth was rinsed with tap water between all the tests. Taste impairment was defined as being unable to identify quinine or NaCl in the whole mouth test.

#### 2.2.3. Covariates

Using prior literature [4], variables that can potentially confound the association between 25(OH)D concentrations and smell and taste assessments were considered. This included gender, age in years divided to groups (40–49, 50–59, 60–69, 70–80 years), race/ethnicity (non-Hispanic White, non-Hispanic Black, Hispanic American or other), education (≤12th grade with no diploma, high-school graduate or equivalent, some college or equivalent degree, college graduate or above). The Income Poverty Ratio (PIR) was used as a proxy measure for socioeconomic status (SES). In America, each state sets the poverty threshold as the minimum amount of annual income people need to pay for essentials. In this study, the respondents were split into three categories based on their PIR presented in percentage, with a higher number indicating higher SES: <185%, 185–350% and >350%.

Smoking status (former, current, or never), alcohol consumption in the past 12 months (never or less than 12 drinks a year, 2 or less drinks a day, 3 drinks, and 4 or more drinks a day), season of examination (November–April or May–October), and Body Mass Index (BMI) (kg/m^2^) calculated as weight in kilograms divided by height in meters squared were noted.

Total energy intake (kcal/day) and the dietary intake of Vitamin D (mcg/day) were calculated based on the 24 h recall dietary interview of day one and included only participants who met the criteria for inclusion based on the NHANES for reliable dietary data [31]. Dietary intake of Vitamin D (mcg/day) was further divided based on its median split (median = 3.1 mcg/day): below 3.1 mcg/day and 3.1 mcg/day and above.

Self-reported chronic diseases were based on the answers to the questions, ‘has a doctor told you had Diabetes? Cardiovascular Disease (CVD)? Asthma? Cancer?’ The diabetes category also included participants with hemoglobin A1c ≥ 6.5%.

### 2.3. Statistical Analysis

All analyses were performed using Stata 15 (StataCorp LP, College Station, TX, USA). Responses coded as “don’t know,” “refused,” or “missing” in the original NHANES were treated as missing. All analyses were performed using the NHANES 2013–2014 sample weights to account for stratification and clustering because of the complex sample design.

Descriptive statistics (sample sizes and weighted proportions) were used to summarize the characteristics of the study sample by the weighted prevalence of taste and smell assessments and to present distributions of main exposure and covariate variables. Several weighted logistic regressions were employed to evaluate the p-value of the overall crude association between the outcomes with the main exposure and with each of the study covariates.

Further, since smell and taste impairments are age related, the age-adjusted associations between the outcomes with the main exposure and each of the study covariates were analyzed using weighted logistic regressions. The multivariable analyses were employed and included weighted logistic regression models to examine the main study’s hypotheses. The initial models first included the main exposure and the target outcomes adjusted for covariates. Some covariates were then eliminated if they did not significantly contribute to the model based on the adjusted Wald test p-value, generating effect estimates of the final model. Adjusted odds ratios (ORs) and their 95% confidence intervals (CIs) were presented.

In addition, two interaction terms comprised of age groups and 25(OH)D concentrations were created, with one term for each outcome, and were tested using the adjusted Wald chi test. The final model was then stratified by age groups if the interaction term was statistically significant. The last analysis step included the chi-square goodness of fit test [32] to evaluate the overall fit of each final model. A p-value>0.05 (not significant) indicated a good model fit, otherwise it was a poor fit. A type I error level of 0.05 was considered significant for testing this study’s hypotheses.

## 3. Results

### 3.1. Study Sample

The study sample is described in Table 1. The final smell sample included 2216 participants and the final taste sample included 2636 participants. Participants were between 40 and 80 years old. Of those, 18.3% reported taste impairment, and 12.2% reported smell impairment. Further, less than 2.0% reported taste and smell impairments, and both impairments were not significantly associated (*p*-value = 0.648) (data are not shown). Among participants with smell impairment, 87.4% failed to recognize the bitter taste, 9% the salt taste, and 3.6% the salt and bitter tastes.

Approximately 20% of the participants suffered from Vitamin D deficiency, with serum 25(OH)D concentrations below 20 ng/mL, and approximately 45% had sufficient Vitamin D, with serum 25(OH)D concentrations above 30 ng/mL. The prevalence of Vitamin D deficiency was higher among participants with smell impairment (24.8%) compared to those with normal smell assessment (19.3%) (*p*-value = 0.123). However, it was similar between participants with vs. without taste impairment, 18.9% vs. 19.2%, respectively (*p*-value = 0.926).

Participants with smell impairment were significantly more elderly, were more likely to be male and non-Hispanic Black, had lower education and lower SES assessed by PIR, had a higher prevalence of chronic diseases such as diabetes, CVD and cancer and consumed less calories and alcohol drinks, compared to their counterparts with normal smell assessment. Additionally, participants with taste impairment were more likely to be non-Hispanic Black, had a higher prevalence of CVD, and consumed more alcohol than their counterparts.

### 3.2. Smell and Taste Impairments across Age Groups

**Figure 1:**
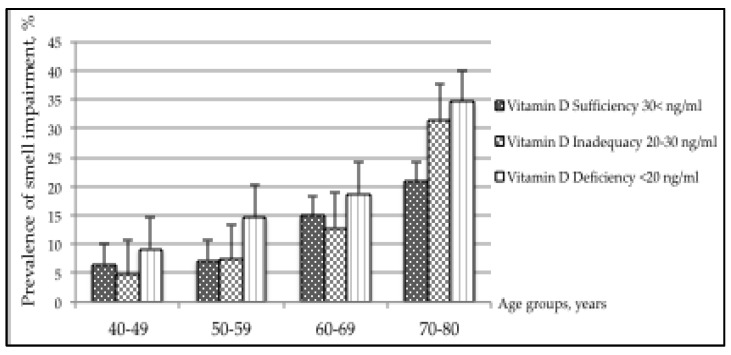
Prevalence of smell impairment across age groups by levels of Vitamin D.

Figure 1 shows the increase in smell impairment prevalence with increased age (*p*-value < 0.001) and with decreased Vitamin D (*p*-value < 0.001). The highest differences of smell impairment prevalence between levels of Vitamin D sufficiency vs. deficiency are shown in age groups 50–59 years (*p*-value < 0.071) and 70–80 years (*p*-value < 0.082). However, the overall interaction term between Vitamin D and age groups was not significant (*p*-value = 0.250) and it was not further examined.

**Figure 2:**
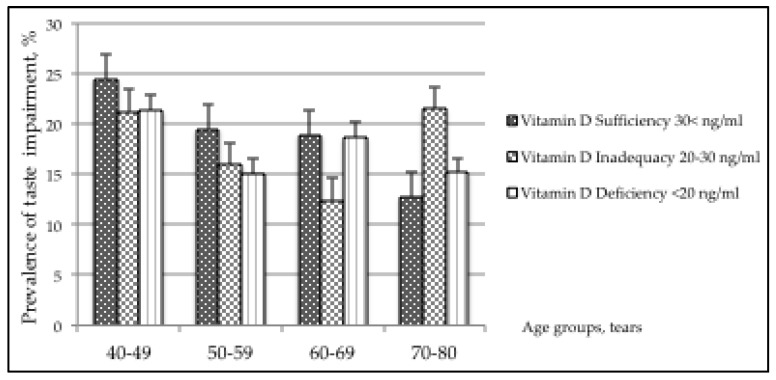
Prevalence of taste impairment across age groups by levels of Vitamin D.

In Figure 2, the prevalence of taste impairment decreases significantly with increased age (*p*-value = 0.049) only among participants with sufficient Vitamin D. A similar trend across age groups was not observed in lower levels of Vitamin D. Only among participants aged 70–80 years with Vitamin D inadequacy was there a significantly higher prevalence of taste impairment (*p*-value = 0.004) compared with those with Vitamin D sufficiency in the same age group. The overall interaction term between Vitamin D and age groups was significant (*p*-value = 0.028) and it was further analyzed.

### 3.3. Smell Impairment and the Association with Vitamin D

The results of the weighted logistic regression models are shown in Table 2. The age-adjusted model indicated that there is a significant relationship between Vitamin D deficiency and smell impairment (OR = 1.76, 95% CI: 1.36–1.37). There were no significant differences between those who smoke vs. never in their ability to correctly identify the tested odorants (OR = 0.88, 95% CI: 0.56–1.38). CVD or diabetes did not significantly affect patient capability to recognize the tested odorants (OR = 1.01, 95% CI: 0.52–1.95; OR = 0.78, 95% CI: 0.58–1.05, respectively). Participants with impaired smell assessment also did not report an increase in BMI, in energy intake, or level of Vitamin D intake compared to those with normal smell assessment.

The final model showed that participants with Vitamin D deficiency were more likely by 39% to report a higher prevalence of smell impairment (OR = 1.39, 95% CI: 1.02–1.89) compared with those with sufficient Vitamin D, after adjusting for significant covariates. In addition, the final model included significant covariates. Females were less likely to report smell impairment than males (OR = 0.48, 95% CI: 0.31–0.73) and non-Hispanic Black were more likely to report smell impairment (OR = 1.57, 95% CI: 1.06–2.32) than non-Hispanic White. Among the examined chronic diseases, only cancer was significantly associated with smell impairment (OR = 2.00, 95% CI: 1.16–3.46). Participants who consumed two or less alcohol drinks a day reported lower prevalence of smell impairment compared to those who drunk less than 12 drinks in the past year (OR = 0.58, 95% CI: 0.40–0.85). The Hosmer and Lemeshow goodness-of-fit test of the final model indicted a good model fit (*p*-value = 0.189).

### 3.4. Taste Impairment and the Association with Vitamin D

Table 3 presents the results of the weighted logistic regression models. The age-adjusted model indicated that there was no significant association between Vitamin D and taste impairment (OR = 0.88, 95% CI: 0.58–1.32). Further examining the target association by including potential confounders in the model generated similar insignificant findings (OR = 0.79, 95% CI: 0.51–1.24).

The final model showed that non-Hispanic Black participants were more likely to report taste impairment (OR = 1.58, 95% CI: 1.07–2.34) than non-Hispanic White participants. Among the examined chronic diseases, only CVD was significantly associated with taste impairment (OR = 1.71, 95% CI: 1.33–2.20). Alcohol drink was significantly associated with taste impairment (OR = 1.43, 95% CI: 1.07–1.89).

The interaction term between Vitamin D and the age group was significant and improved the Hosmer and Lemeshow goodness-of-fit test to be significant (*p*-value = 0.920), indicating a good fit of the final model. Therefore, the final model was then further stratified by age groups (Table 4).

Table 4 shows four age stratum of the adjusted target association. Only among participants in the age group 70–80 years, Vitamin D inadequacy was significantly associated with taste impairment (OR = 1.96, 95% CI: 1.35–1.85). In contrast, such a significant finding was not observed within the same age stratum at 25(OH)D concentrations below <20 ng/mL (OR = 1.15, 95% CI: 0.45–2.99).

## 4. Discussion

To the best of the author’s knowledge, this is the first epidemiological study that examined the relationships between Vitamin D and smell and taste impairments among national representative adult samples from the US. This study sheds light on the potential mechanism that may link Vitamin D deficiency and age-adjusted smell impairment. Specifically, this study showed that participants with Vitamin D deficiency, serum 25(OH)D concentrations below 20 ng/mL, were more likely by 39% to report smell impairment compared to those with sufficient Vitamin D, serum 25(OH)D concentrations above 30 ng/mL, after adjusting for significant confounders such as age, gender, race/ethnicity, and history of cancer.

This study also showed that only participants aged 70–80 years with Vitamin D adequacy, serum 25(OH)D concentrations of 20–30 ng/mL, were more likely by 96% to report taste impairment compared to participants with sufficient Vitamin D after adjusting for significant confounders such as age, gender, race/ethnicity, CVD and BMI. However, such a significant relationship between Vitamin D and taste impairment was not observed among participants younger than 70 years of age. More studies with improved methods are needed to explore such a potential association.

In the literature, limited studies have investigated Vitamin D deficiency and its relationships with smell and showed similar findings. For example, a study of a few reported cases showed that olfactory dysfunction has subjectively been detected in adult patients with Vitamin D deficiency and it was improved with increasing serum Vitamin D [22]. Another study found that low serum (25(OH)D) concentrations were independently associated with olfactory dysfunction among Parkinson’s disease patients [33]. However, similar studies regarding Vitamin D and taste dysfunction were not found in the literature.

Ample epidemiological studies showed that Vitamin D deficiency is associated with brain health including cognitive function, neuropsychiatric disorders [34] and mood regulation [7,8,9]. This stems from the fact that Vitamin D receptors are widely spread throughout the central and peripheral nervous systems and have an impact on various neurotransmitters [12,13,22,34]. Some animal studies have shown that the rat olfactory system contains numerous target sites of Vitamin D receptor [17,35], which emphasizes the unique functional importance of 1,25-dihydroxy Vitamin D3 in olfactory function [33]. Since Vitamin D functions as a neurosteroid hormone in the brain, spinal cord, and olfactory system [36], a lack of Vitamin D may lead not only to the neurological decline of the central and peripheral nervous system but also of the cranial nerves, causing a reduction in olfactory perception [22,37], which may explain the significant finding observed in the current study.

However, this study did not find a significant relationship between Vitamin D deficiency and taste impairment. It seems that although smell and taste are often grouped, the brain processes them differently, and so each possesses unique mechanisms and associated causes [2]. This was also confirmed in this study, as no significant relationship between smell and taste impairments was observed, and therefore, participants in this sample with smell impairment did not necessarily have taste impairment. Taste receptors are associated with cranial nerves VII, IX, and X, which are linked to the gustatory cortex of the brain [38], while smell receptors are associated with cranial nerve I, which is linked to the olfactory cortex [39]. Since only two tastes were examined in this study, as opposed to eight odorants in the smell examination, subsequent studies might need to add more tasting such as sweet, sour, and umami when examining the association with Vitamin D deficiency among adults.

People with smell or taste dysfunction tend to report common symptoms such as a loss of appetite, or changes in food intake including meats, fresh fruits, coffee, eggs, and carbonated beverages [40,41]. In the current sample, however, participants with smell or taste impairment did not report differences in their calorie intakes, in their dietary intake of Vitamin D or in their BMI compared to those with normal assessments of smell and taste. This might be due to compensating with other food intakes, thus maintaining the total energy intake. Other changes related to culinary habits are also common when a decrease in smell and taste occurs, such as increased salt, sugar, and fat use, which could also cause other health issues such as hypertension, diabetes, and CVD, but were not assessed in this study [40,41].

The present study had several strengths. This study utilized data from the NHANES, which employed standardized protocols and rigorous quality control in data collection and reporting. The large sample of individuals is representative of the US population; therefore, these results can be applied to the US population, but they may not be applied to other populations who may have different predispositions for smell and taste impairments and Vitamin D metabolism. In addition, Vitamin D was measured via serum 25(OH)D concentrations using tandem LC–MS/MS, which is considered a standard methodology that allows comparison to other national surveys. Smell assessments employed a relatively valid number of odorants and impairment corresponds to the UPSIT [29].

The NHANES has a large, rich and unique database that was used to examine the target hypotheses while controlling for well-established confounders associated with smell and taste impairments such as age, gender, race/ethnicity [4], and associated chronic diseases including cancer, diabetes [42], CVD and asthma to ensure an accurate and valid estimation of the findings. Individuals who had reported problems with their nose, such as nasal/sinus congestion were excluded to properly assess primary exposure with age-related smell loss.

To the author’s knowledge, this is the first large-scale well-designed epidemiological study to explore the association between Vitamin D and smell and taste impairments using population-based adult samples. The significant findings indicate a possible mechanism between Vitamin D and the olfactory system across age groups, which bridges a gap in knowledge in this field.

The present study also had several limitations. A cross-sectional design was used to evaluate the relationship between serum 25(OH)D concentrations and current smell and taste status. However, in conclusion, temporality and causality relationships are impossible. Although a representative sample of adults aged 40 and over living in the US was used, the findings should be extrapolated with caution outside these limits.

The assessment of smell and taste impairments was measured only one time, and may not be reproducible over longer period. In the taste assessment, the NHANES only utilized two tastings, bitter and salt, which might not be sensitive enough to capture age-related taste loss. Further, the type of smell test used by the NHANES only measures the ability to identify a particular odor but does not measure sensitivity or the threshold to certain smells. Therefore, this study did not differentiate between smell disorders caused by a decrease in the ability to smell or changes in the way a person perceives odors.

There are additional age-related health conditions that may directly cause smell and taste impairments such as Parkinson’s disease, Alzheimer’s disease, and hypothyroidism [33,40]. Furthermore, data on medications (i.e., some antibiotics and antihistamines) that can potentially influence taste impairment were not included, which might also affect serum Vitamin D [5].

## 5. Conclusions

The key finding of this study was that Vitamin D might have a significant role in the human olfactory system, and deficiency in Vitamin D might increase age-related smell impairment among adults. Additionally, Vitamin D might also play a vital role in the human gustatory system, since taste impairment was found to be significantly associated with Vitamin D inadequacy in the elderly aged 70–80 years. To elucidate the causal links between Vitamin D and smell and taste status, more studies are warranted, as such knowledge is essential to reduce these public health concerns. Once such relationships are determined, improving Vitamin D deficiency can mitigate loss in age-related smell and taste status among adults.

## Figures and Tables

**Table 1:** Summary statistics of the study sample according to smell and taste assessments in American adults aged 40–80 years, the NHANES 2013–2014.

	Taste Assessment *n* = 2636		*p*-Value	Smell Assessment *n* = 2216		*p*-Value
	Normal	Impairment		Normal	Impairment	
**Vitamin D (25(OH)D) (ng/mL)**	***n* = 2142 (81.7%)**	***n* = 494 (18.3%)**	0.926	***n* = 1866 (87.8%)**	***n* = 350 (12.2%)**	0.123
Sufficiency >30	848(45.4)	188(47.0)		723(44.9)	127(45.7)	
Inadequacy 20–30	770(35.4)	178(34.1)		673(35.8)	124(29.5)	
Deficiency <20	524(19.2)	128(18.9)		470(19.3)	99(24.8)	
Gender			0.760			0.004
Male	1032(48.8)	234(48.0)		1016(53.4)	216(61.3)	
Female	1110(51.2)	260(52.0)		850(46.6)	134(38.7)	
Age groups (years)			0.114			<0.001
40–49	577(27.6)	163(35.6)		600(33.0)	44(16.5)	
50–59	557(30.2)	131(27.9)		500(30.7)	67(22.1)	
60–69	539(24.0)	110(21.7)		459(22.5)	105(28.4)	
70–80	469(18.2)	90(14.8)		307(13.8)	134(33.0)	
Race/ethnicity			0.023			0.037
Non-Hispanic White	1064(75.3)	240(72.9)		879(73.8)	137(66.1)	
Hispanic American	428(9.8)	99(10.0)		391(10.4)	77(12.5)	
Non-Hispanic Black	386(8.6)	117 (12.5)		364(9.5)	85(13.1)	
Other race	264(6.3)	38(4.6)		232(6.2)	51(8.3)	
Education			0.557			<0.001
≤12th grade with no diploma	404(13.0)	93(12.5)		338(11.8)	113(24.5)	
High-school graduate/equivalent	469(21.4)	134(25.0)		423(22.0)	79(20.7)	
Some college/equivalent	655(31.5)	145 (30.8)		566(31.2)	80(27.9)	
College graduate and above	614(34.1)	122(31.8)		539(35.0)	78(26.9)	
PIR (percentiles)			0.166			0.008
<185%	831(27.3)	210(28.4)		709(26.5)	161(32.9)	
185–350%	519(23.6)	123(27.7)		451(23.6)	93(26.6)	
350%<	792(49.1)	161(43.9)		706(49.9)	96(27.5)	
Smoking status			0.355			0.378
Never	1141(53.5)	235(50.4)		1,028(56.2)	191(53.6)	
Former	633(30.1)	149(29.5)		527(28.3)	109(33.4)	
Current	368(16.4)	110(20.1)		311(15.5)	50(13.0)	
Alcohol (drinks/day)			0.014			0.004
Never/< 12 drinks a year	735(28.4)	156(25.0)		584(25.3)	172(40.7)	
≤2	674(32.1)	136(28.9)		608(33.2)	87(24.9)	
≥3	383(21.9)	90(22.5)		344(22.3)	50(20.6)	
≥4	350(17.6)	112(23.7)		330(19.2)	41(13.7)	
Season of examination *			0.085			0.909
November–April	1062(45.4)	226(41.1)		910(46.6)	157(43.1)	
May–October	1080(54.6)	268(58.1)		956(56.4)	193(56.9)	
BMI (kg/m^2^) **	29.6(6.7)	29.5(7.5)	0.847	29.4(6.7)	30.2(8.1)	0.215
Chronic diseases						
Diabetes	446(16.7)	103(18.8)	0.465	365(15.8)	87(21.0)	0.038
CVD	124(5.4)	34(7.4)	0.026	87(4.3)	29(7.6)	0.021
Cancer	313(17.1)	55(13.5)	0.221	220(13.3)	58 (24.9)	0.002
Asthma	307(14.1)	79(16.0)	0.406	251(13.4)	47(16.6)	0.227
Dietary intake of Vitamin D(mcg/day)			0.546			0.981
Intake <3.1	1069(49.2)	252(50.6)		933(50.1)	183(50.2)	
Intake ≥3.1	1073(50.8)	242(49.4)		933(49.9)	167(49.8)	
Energy intake (kcal/day) **	2085(922)	2084(951)	0.987	2094(886)	1930(974)	0.044

* Season of examination of serum 25(OH)D concentrations. ** Mean (SD). NHANES, National Health and Nutrition Examination Survey; 25(OH)D, serum 25-hydroxyvitamin D; BMI, Body Mass Index; PIR, Poverty Income Ratio; CVD, Cardiovascular Disease; ng/mL, nanograms per milliliter; mcg, micrograms; m, meter. The values are the percent or mean and are all weighted from the NHANES 2013–2014 sampling weights. For main exposure and covariate presented in percent, the percentages add up to 100% for a column.

**Table 2:** Adjusted associations between Vitamin D (25(OH)D) and smell impairment in American adults aged 40–80 years (*n* = 2216), the NHANES 2013–2014.

Vitamin D (25(OH)D) (ng/mL)	Age-Adjusted OR Model	*p*-Value	Adjusted OR Initial Model	*p*-Value	Adjusted OR Final Model	*p*-Value
	OR (95% CI)		OR (95% CI)		OR (95% CI)	
Sufficiency >30	1.00		1.00		1.00	
Inadequacy 20–30	1.05(0.70–1.59)	0.796	0.85(0.56–1.29)	0.406	0.87(0.59–1.30)	0.482
Deficiency <20	**1.79(1.36–2.37)**	**0.000**	**1.36(1.01–1.82)**	**0.042**	**1.39(1.02–1.89)**	**0.038**
Gender						
Male	1.00		1.00		1.00	
Female	**0.52(0.36–0.76)**	**0.002**	**0.40(0.24–0.67)**	**0.002**	**0.48(0.31–0.73)**	**0.002**
Age groups (years)						
40–49	1.00		1.00		1.00	
50–59	1.44(0.76–2.73)	0.247	1.39(0.70–2.75)	0.326	1.34(0.68–2.64)	0.370
60–69	**2.53(1.36–4.71)**	**0.006**	**2.30(1.15–4.64)**	**0.022**	**2.36(1.17–4.76)**	**0.020**
70–80	**4.78(2.93–7.81)**	**0.000**	**4.28(2.25–8.16)**	**0.000**	**4.30(2.41–7.68)**	**0.000**
Race/ethnicity						
Non-Hispanic White	1.00		1.00		1.00	
Hispanic American	**1.80(1.26–2.57)**	**0.003**	1.37(0.92–2.06)	0.113	1.35(0.87–2.10)	0.171
Non-Hispanic Black	**1.84(1.41–2.38)**	**0.000**	1.51(0.99–2.29)	0.053	**1.57(1.06–2.32)**	**0.027**
Other race	**1.91(1.19–3.07)**	**0.011**	**2.15(1.29–3.59)**	**0.006**	**2.96(1.21–3.17)**	**0.010**
Education						
≤12th grade no diploma	1.00		1.00		**1.00**	
High-school graduate	**0.46(0.27–0.76)**	**0.005**	**0.47(0.27–0.81)**	**0.009**	**0.49(0.29–0.84)**	**0.012**
Some college	**0.43(0.33–0.57)**	**0.000**	**0.44(0.32–0.62)**	**0.000**	**0.47(0.33–0.65)**	**0.000**
College graduate/above	**0.40(0.27–0.60)**	**0.000**	**0.44(0.27–0.71)**	**0.002**	**0.44(0.27–0.71)**	**0.003**
PIR (percentiles)						
<185%	1.00		1.00		--	--
185%-350%	0.98(0.71–1.35)	0.894	1.20(0.90–1.60)	0.194	--	--
350%<	**0.66(0.49–0.90)**	**0.011**	0.92(0.66–1.28)	0.602	--	--
Smoking status						
Never	1.00		1.00		--	--
Former	1.05(0.69–1.59)	0.815	0.89(0.58–1.37)	0.571	--	--
Current	1.13(0.70–1.82)	0.597	0.88(0.56–1.38)	0.544	--	--
Alcohol (drinks/day)						
Never/ <12 drinks a year	1.00		1.00		1.00	
≤2	**0.58(0.40–0.84)**	**0.006**	**0.59(0.40–0.88)**	**0.012**	**0.58(0.40–0.85)**	**0.009**
≥3	0.81(0.56–1.17)	0.242	0.88(0.61–1.28)	0.489	0.82(0.55–1.20)	0.280
≥4	0.63(0.33–1.19)	0.145	0.67(0.35–1.27)	0.202	0.61(0.31–1.20)	0.144
Season of examination *						
November–April	1.00		1.00		--	--
May–October	0.95(0.67–1.34)	0.751	1.12(0.84–1.49)	0.408	--	--
BMI (kg/m^2^)	1.02(0.99–1.05)	0.125	1.02(0.99–1.05)	0.084	--	--
Chronic diseases						
Diabetes	1.18(0.84–1.66)	0.317	0.78(0.58–1.05)	0.098	--	--
CVD	1.10(0.60–2.00)	0.752	1.01(0.52–1.95)	0.982	--	--
Cancer	1.83(1.94–2.57)	0.083	**2.06(1.22–3.45)**	**0.010**	**2.00(1.16–3.46)**	**0.016**
Asthma	1.28(0.83–1.98)	0.251	1.46(0.95–2.22)	0.073	--	--
Dietary intake of Vitamin D (mcg/day)						
<3.1	1.0		1.0		--	--
≥3.1	0.93(0.66–1.32)	0.669	0.99(0.68–1.46)	0.996	--	--
Energy intake (kcal/day)	0.99(0.99–1.00)	0.299	0.99(0.99–1.00)	0.112	--	--

–, not included in the final model. * Season of examination of 25(OH)D concentrations. NHANES, National Health and Nutrition Examination Survey; 25(OH)D, serum 25-hydroxyvitamin D; BMI, Body Mass Index; PIR, Poverty Income Ratio; CVD, Cardiovascular Disease; ng/mL, nanograms per milliliter; mcg, micrograms; m, meter. Hosmer and Lemeshow goodness-of-fit test: F-adjusted test statistic = 1.050 *p*-value = 0.189. Interaction term between age groups and serum 25(OH)D concentrations adjusted based on the final model, *p*-value= 0.492. Statistically significant values (*p*-Value < 0.05) are given in bold.

**Table 3:** Adjusted associations between Vitamin D (25(OH)D) and taste impairment among American adults aged 40–80 years (*n* = 2636), the NHANES 2013–2014.

Vitamin D (25(OH)D) (ng/mL)	Age-Adjusted OR Model	*p*-Value	Adjusted OR Final Model	*p*-Value
	OR (95% CI)		OR (95% CI)	
Sufficiency > 30	1.00		1.00	
Inadequacy 20–30	0.88(0.60–1.28)	0.466	0.86(0.59–1.26)	0.412
Deficiency <20	0.88(0.58–1.32)	0.515	0.79(0.51–1.24)	0.288
Gender				
Male	1.00		--	--
Female	1.03(0.84–1.26)	0.739	--	--
Age groups (years)				
40–49	1.00		1.00	
50–59	0.72(0.50–1.03)	0.069	0.71(0.50–1.00)	0.055
60–69	0.70(0.49–1.00)	0.051	0.69(0.47–1.01)	0.056
70–80	0.63(0.43–0.92)	0.021	**0.61(0.42–0.89)**	**0.014**
Race/ethnicity				
Non-Hispanic White	1.00		1.00	
Hispanic American	0.96(0.63–1.46)	0.836	1.01(0.66–1.55)	0.953
Non-Hispanic Black	1.44(1.00–2.08)	0.050	**1.58(1.07–2.34)**	**0.024**
Other race	0.70(0.40–1.21)	0.187	0.76(0.42–1.35)	0.319
Education				
≤12th grade	1.00		--	--
High-school graduate	1.21(0.81–1.81)	0.326	--	--
Some college	1.02(0.68–1.50)	0.936	--	--
College graduate or above	0.96(0.62–1.48)	0.934	--	--
PIR (percentiles)				
<185%	1.00		--	--
185–350%	1.12(0.78–1.62)	0.518	--	--
350%<	0.84(0.60–1.20)	0.328	--	--
Smoking status				
Never	1.00		--	--
Former	1.10(0.80–1.51)	0.524	--	--
Current	1.25(0.89–1.76)	0.181	--	--
Alcohol (drinks/day)				
Never/< 12 drinks a year	1.00		1.00	
≤2	0.98(0.72-1.37)	0.937	0.99(0.72–1.39)	0.995
≥3	1.12(0.73-1.72)	0.578	1.15(0.74–1.77)	0.508
≥4	1.39(1.04-1.86)	0.029	**1.43(1.07–1.89)**	**0.018**
Season of examination *				
November–April	1.00		--	--
May–October	1.22 (0.98–1.50)	0.063	--	--
BMI (kg/m^2^)	0.99(0.97–1.03)	0.811	--	--
Chronic diseases				
Diabetes	1.24(0.82–1.83)	0.288	--	--
CVD	1.67(1.24–2.24)	0.002	**1.71(1.33–2.20)**	**0.000**
Cancer	0.83(0.48–1.45)	0.494	--	--
Asthma	1.17(0.80–1.69)	0.395	--	--
Dietary intake of Vitamin D (mcg/day)				
<3.1	1.00		--	--
≥3.1	0.97(0.80–1.16)	0.712	--	--
Energy intake (kcal/day)	0.99(0.99–1.00)	0.789	--	--

–, not included in the final model. * Season of examination of serum 25(OH)D concentrations. NHANES, National Health and Nutrition Examination Survey; 25(OH)D, serum 25-hydroxyvitamin D; BMI, Body Mass Index; PIR, Poverty Income Ratio; CVD, Cardiovascular Disease; ng/mL, nanograms per milliliter; mcg, micrograms; m, meter. Hosmer and Lemeshow goodness-of-fit test: F-adjusted test statistic = 0.364 *p*-value =0.920. Interaction term between age groups and Vitamin D adjusted based on the final model, *p*-value= 0.024. Statistically significant values (*p*-Value < 0.05) are given in bold.

**Table 4:** Adjusted associations between Vitamin D (25(OH)D) and taste impairment by age groups in American adults (*n* = 2636), the NHANES 2013–2014.

Vitamin D (25(OH)D) (ng/mL)	Crude OR	*p*-Value	Adjusted OR*	*p*-Value
Age group 40–49 years, *n* = 740	OR (95% CI)		OR (95% CI)	
Sufficiency >30	1.00		1.00	
Inadequacy 20–30	0.83(0.43–1.62)	0.562	0.83(0.45–1.55)	0.542
Deficiency <20	0.84(0.39–1.82)	0.633	0.74(0.37–1.46)	0.357
**Age group 50–59 years, *n* = 688**				
Sufficiency >30	1.00		1.00	
Inadequacy 20–30	0.78(0.36–1.69)	0.508	0.76(0.35–1.67)	0.474
Deficiency <20	0.73(0.31–1.74)	0.453	0.62(0.25–1.52)	0.272
**Age group 60–69 years, *n* = 649**				
Sufficiency >30	1.00		1.00	
Inadequacy 20–30	0.61(0.33–1.09)	0.088	0.59(0.32–1.09)	0.088
Deficiency <20	0.98(0.52–1.88)	0.955	0.95(0.46–1.93)	0.871
**Age group 70–80 years, *n* = 559**				
Sufficiency >30	1.00		1.00	
Inadequacy 20–30	**1.88(1.27–2.78)**	0.004	**1.96(1.35–2.85)**	**0.002**
Deficiency <20	1.22(0.51–2.93)	0.635	1.15(0.45–2.98)	0.753

*Adjusted Odds Ratio (OR)- based on the final model covariates: race/ethnicity, alcohol and CVD. NHANES, National Health and Nutrition Examination Survey; 25(OH)D, serum 25-hydroxyvitamin D ng/mL, nanograms per milliliter. Statistically significant values (*p*-Value < 0.05) are given in bold.
